# Artificial Intelligence–Derived Electrocardiographic Age Predicts Mortality in Adults With Congenital Heart Disease

**DOI:** 10.1016/j.jacadv.2025.101777

**Published:** 2025-05-14

**Authors:** Scott Anjewierden, Donnchadh O'Sullivan, Kathryn E. Mangold, Itzhak Zachi Attia, Francisco Lopez-Jimenez, Paul A. Friedman, Alexander C. Egbe, Heidi M. Connolly, William R. Miranda, Samuel J. Asirvatham, Jennifer Dugan, Katia Bravo-Jaimes, Talha Niaz, Malini Madhavan, Luke J. Burchill

**Affiliations:** a Department of Pediatric and Adolescent Medicine, Division of Pediatric Cardiology, Mayo Clinic, Rochester, Minnesota, USA; b Department of Cardiovascular Medicine, Mayo Clinic, Rochester, Minnesota, USA; c Department of Cardiovascular Medicine, Mayo Clinic, Jacksonville, Florida, USA

**Keywords:** adults with congenital heart disease, artificial intelligence, congenital heart disease, mortality, risk factor

## Abstract

**Background:**

Artificial intelligence (AI) can be used to estimate age from the electrocardiogram (AI-ECG age). The difference between AI-ECG age and chronological age (delta-age) is an independent predictor of mortality in the general population.

**Objectives:**

The purpose of this study was to assess the relationship between delta-age and mortality among adults with congenital heart disease (ACHD).

**Methods:**

A previously validated neural network was used to analyze standard digital 12-lead ECGs in a cohort of ACHD (age >18 years) between 1992 and 2023. A single ECG from each patient, collected during the first visit to the ACHD clinic, was analyzed to compute the delta-age. The relationship between the delta-age and mortality was evaluated using Cox proportional hazard models adjusting for influential clinical factors.

**Results:**

Of 5,780 subjects tested (50% females), the mean chronological age was 39.1 ± 15.0 years. AI-ECG age was 52.3 ± 16.6 years. CHD complexity was classified as mild, moderate, and severe in 7.4%, 73.9%, and 18.7% of patients, respectively. Patients with severe CHD had the highest median delta-age of 15.8 (IQR: 3.5-31.2) years followed by moderate 11.5 (IQR: 3.5-21.3) years and simple 6.7 (IQR: 0.3-14.2) years. During a median follow-up of 6.4 years (IQR: 1.58-13.7 years), 839 (14.5%) patients died. After adjusting for chronologic age, CHD complexity, and other clinical variables, delta-age was associated with increased mortality risk (HR: 1.06 [1.03-1.09] per 5-year increment in delta-age, *P* < 0.05).

**Conclusions:**

Delta-age, the difference between AI-ECG and chronological age, is an independent predictor of all-cause mortality in ACHD.

Advances in medical and surgical care of children born with congenital heart disease (CHD) has dramatically improved their survival.^1^ Over 95% of children with CHD are expected to survive to adulthood, and 75% of these survivors live to be over 60 years of age.^2^ As adults with CHD (ACHD) age, they have greater health care utilization, morbidity, and mortality at a younger age than non-CHD adults.^3^ There is a need for a paradigm shift in risk assessment of patients with ACHD where traditional measures such as chronological age will systematically underestimate the risk of morbidity and mortality.

Artificial intelligence (AI)-based algorithms promise to deliver enhanced diagnostic and prognostic capabilities in medicine. We have previously reported the application of convolutional neural networks (CNN) in electrocardiogram (ECG) to estimate age in adults in the general population with accuracy.^4^ The AI-estimated ECG-age correlates well with the occurrence of medical comorbidities and mortality, suggesting that the ECG-age is a better assessment of physiology than chronological age.^4^, ^5^, ^6^ ACHD is associated with accelerated aging and earlier occurrence of cardiovascular comorbidities such as diabetes, heart failure, and arrhythmias, all of which influence the ECG signatures of different ACHD conditions.^7^^,^^8^ We hypothesized that AI-enhanced interpretation of ECG in ACHD will yield an estimation of the biological age that correlates with comorbidities and mortality.

## Methods

### Study population and data sources

This retrospective cohort study included adult patients (age >18 years) evaluated at the Mayo Clinic for Adult Congenital Heart Disease Clinic between the years 1992 and 2023. Patients had an ECG recorded during the same year as their initial visit. Those that had missing data (either clinical information or ECG data) were excluded. All patients consented for the use of their medical records for research purposes, and the study was approved by the Mayo Clinic Institutional Review Board.

Clinical data were collected from the electronic health record. The ECG data for each patient were collected using a Marquette ECG machine (GE Healthcare). Data were stored using the MUSE data-management system, and all ECGs were collected with a sampling rate of 500 Hz. Mortality data were obtained by manual extraction from the Mayo Clinic electronic medical records and from the LexisNexis Accurint (New York, NY) database, which includes over 33 billion records across more than 8,000 different data sources.^9^

### Baseline data and outcomes

The AI-ECG-estimated age was obtained using a CNN, whose input was the raw ECG data with an output of AI-ECG-estimated age as a continuous number. The development, training, and architecture of our algorithm has been previously described and validated in the general adult population.^4^ While not publicly available, this algorithm is available to external research groups through collaborative projects subject to institutional approval and data-use agreements. We used the previously described network without any additional retraining or modifications. To examine the relationship between AI-ECG age and chronological age, we evaluated the “delta-age” for each patient, which was defined as the difference between AI-ECG-estimated age and the patient's chronological age at the time of the ECG. A single ECG, collected during the year of the first visit to the ACHD clinic, was analyzed to compute the delta-age. Using this definition, a positive delta-age indicates AI-ECG-estimated age higher than the subject's chronological age. We then evaluated the relationship between this delta-age and risk of mortality.

Other baseline clinical variables were collected at the time of their first visit ACHD clinic visit as well. CHD complexity was defined as mild, moderate, or severe according to the Bethesda classification system.^10^ ACHD patients were further grouped into 6 categories: single ventricle, systemic right ventricle, left heart lesions, right heart/conotruncal lesions, shunts, and others. For survival analyses, follow-up time was defined as the time from the first ACHD clinic visit to the most recent clinic visit (virtual or in-person) recorded in the Mayo Clinic health system.

### Statistical methods

Baseline clinical data and characteristics for study subjects were summarized with frequencies and percentages or mean ± SD as appropriate. Delta-age was evaluated across different CHD complexity. The distribution of delta-age in specific categories of ACHD lesions, that is, single ventricle, systemic right ventricle, left heart lesions, right heart/conotruncal lesions, shunts, and others, was also evaluated. A multivariable logistic regression analysis was performed to assess the association between clinical and demographic variables and deviation of the delta-age from the mean by more than 1 SD in either direction. For model development, clinical and demographic variables felt to be clinically relevant within the ACHD population were included. Multicollinearity was assessed using the variance inflation factor with minimal multicollinearity among predictors. Model performance was evaluated using McFadden's pseudo R^2^.

The correlation between delta-age and mortality was investigated. Delta-age was evaluated both as a continuous variable and as a categorical variable. For the latter, delta-age was categorized into 3 groups after computing the mean ± SD of the delta-age for the entire cohort. The groups included those with delta-age <1 SD from mean, within ±1 SD, and >1 SD from the mean. Kaplan-Meier curves were plotted, using the log-rank test to evaluate significant differences between groups. Cox proportional hazards models were developed to predict mortality, adjusting for chronological age, sex, CHD complexity, and other baseline clinical factors significantly associated with mortality on univariable analysis (delta-age, history of atrial arrhythmia, sustained ventricular tachycardia, NYHA functional class, hypertension, type 2 diabetes mellitus, smoking history, coronary artery disease, chronic kidney disease, cirrhosis, stroke, anemia, polycythemia). The proportionality assumption for the Cox proportional hazards assumption was assessed graphically for all models and fulfilled. We further evaluated the correlation between delta-age and mortality by plotting polynomial smoothing splines to show the graphical relationship. A Cox proportional hazards model was fitted using natural splines with 4 degrees of freedom to capture the nonlinear relationship between delta-age and mortality. Predicted hazard ratios and their 95% confidence intervals were generated over a range of delta-age from −10 to 40 years.

These analyses were conducted with the python programming language (Python Software Foundation) version 3.11.5 using the pandas, NumPy, scikit-learn, matplotlib, seaborn, and lifelines packages and R version 4.0.3 (R Foundation for Statistical Computing, Vienna, Austria) in the RStudio environment (RStudio Team, 2021).

## Results

### AI-ECG estimated age in ACHD

The cohort consisted of 5,780 ACHD patients with a mean age of 39.1 (±15.0) years, and 50% were male. CHD complexity was mild, moderate, and severe in 7.4%, 73.9%, and 18.7% of patients, respectively. The baseline characteristics of the cohort stratified by CHD complexity are presented in Table 1.

**Table 1:** Baseline Characteristics of Adults With Congenital Heart Disease Stratified by CHD Complexity

	Full Cohort (N = 5,780)	Mild CHD Complexity (n = 427)	Moderate CHD Complexity (n = 4,270)	Severe CHD Complexity (n = 1,083)
Demographics				
Chronological age, y	39.1 ± 15.0	43.2 ± 16.4	40.1 ± 15.2	33.5 ± 12.4
Male	2,912 (50.4%)	177 (41.5%)	2,178 (51.0%)	557 (51.4%)
AI-ECG age, y	52.3 ± 16.6	50.9 ± 15.5	52.8 ± 16.1	50.6 ± 18.8
Delta-age, y	13.2 ± 14.6	7.7 ± 11.5	12.7 ± 13.6	17.1 ± 18.2
Clinical history				
Atrial arrhythmia	1,439 (24.9%)	19 (4.4%)	991 (23.2%)	429 (39.6%)
Sustained VT	128 (2.2%)	2 (0.5%)	104 (2.4%)	22 (2.0%)
NYHA functional class				
I	3,854 (66.7%)	297 (69.6%)	2,600 (60.9%)	927 (85.6%)
II	1,124 (19.4%)	71 (16.6%)	996 (23.3%)	87 (8.0%)
III	689 (11.9%)	48 (11.2%)	579 (13.6%)	62 (5.7%)
IV	113 (2.0%)	11 (2.6%)	95 (2.2%)	7 (0.6%)
Hypertension	1,504 (26.0%)	134 (31.4%)	1,267 (29.7%)	103 (9.5%)
Type 2 diabetes mellitus	418 (7.2%)	56 (13.1%)	326 (7.6%)	36 (3.3%)
Smoking history	986 (17.1%)	102 (23.9%)	778 (18.2%)	106 (9.8%)
Coronary artery disease	335 (5.8%)	27 (6.3%)	292 (6.8%)	16 (1.5%)
Chronic kidney disease	236 (4.1%)	23 (5.4%)	174 (4.1%)	39 (3.6%)
Cirrhosis	93 (1.6%)	4 (0.9%)	5 (0.1%)	84 (7.8%)
Stroke	308 (5.3%)	24 (5.6%)	202 (4.7%)	82 (7.6%)
Anemia	1,427 (24.7%)	84 (19.7%)	1,149 (26.9%)	194 (17.9%)
Polycythemia	529 (9.2%)	34 (8.0%)	234 (5.5%)	261 (24.1%)

Values are (%) or mean ± SD.

AI-ECG-age = artificial intelligence–enabled ECG age; delta-age: difference between AI-ECG and chronological age; VT = ventricular tachycardia.

**Figure 1:**
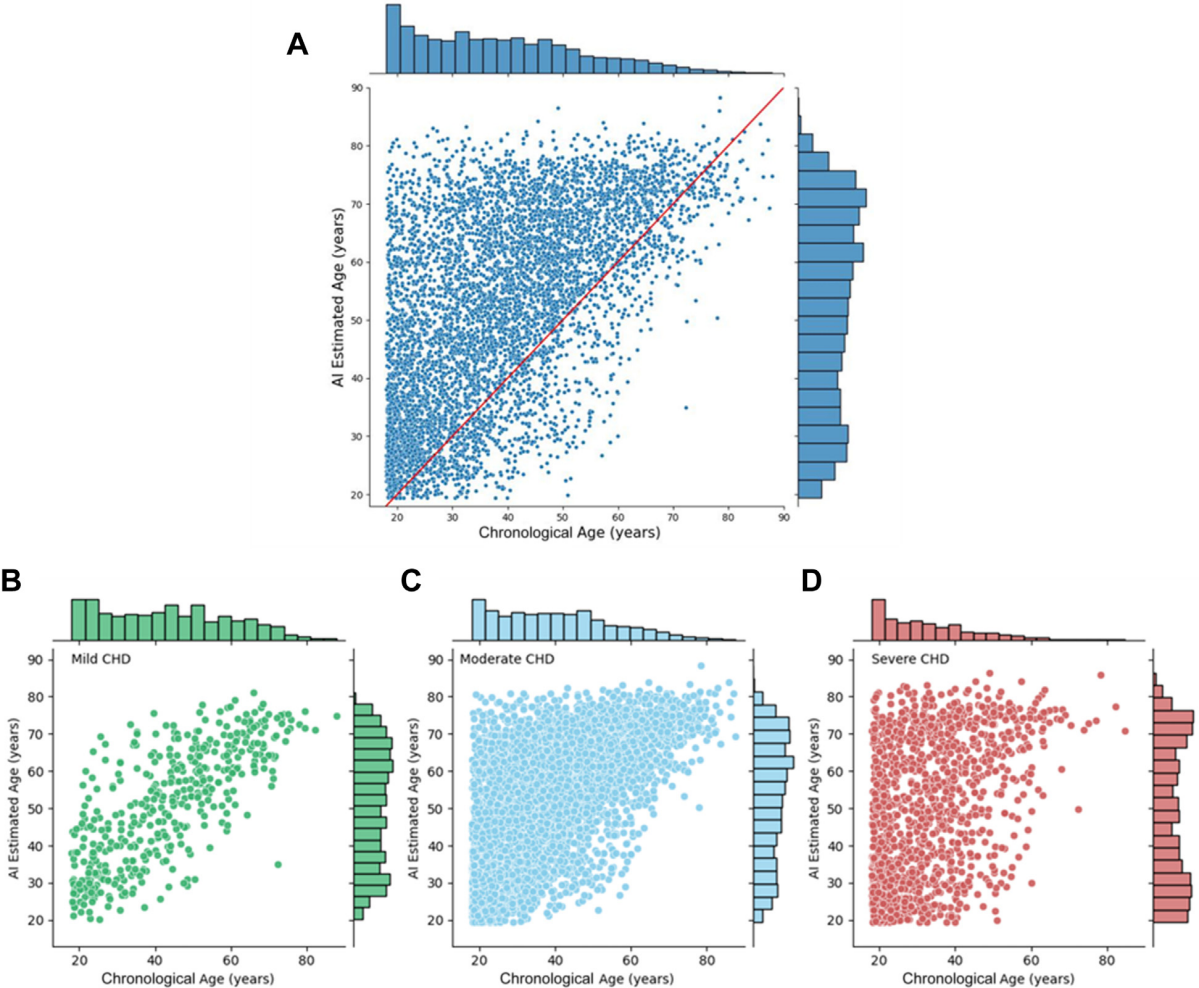
Comparison of Chronological Age and AI-ECG-Estimated Age in Adults With CHD Chronological age compared to AI-ECG estimated age in (A) the full cohort, (B) patients with mild CHD complexity, (C) moderate CHD complexity, and (D) severe CHD complexity. AI-ECG = artificial intelligence enabled electrocardiogram; CHD = congenital heart disease.

**Figure 2:**
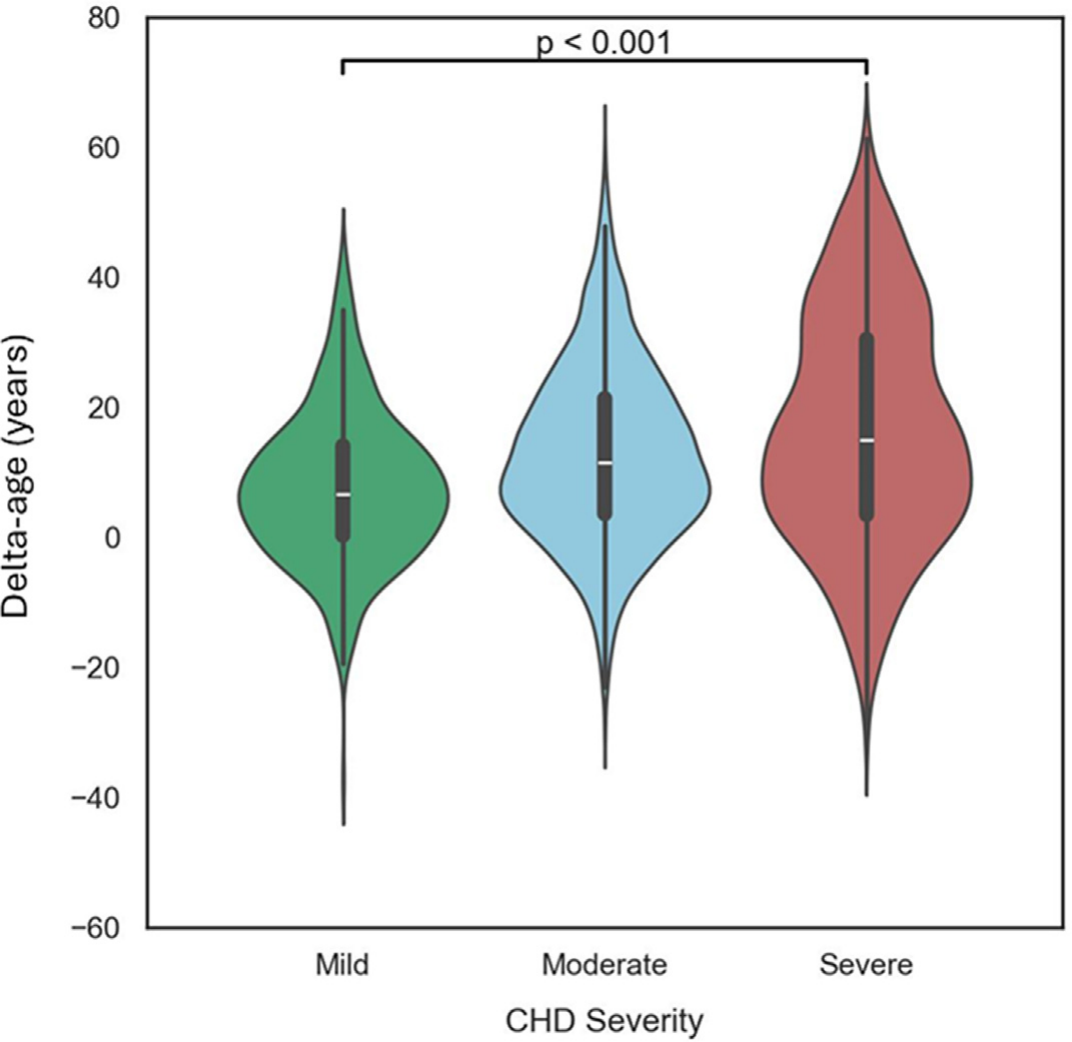
Delta-Age by Degree of Congenital Heart Disease Severity Increase in congenital heart disease severity, as defined by the Bethesda criteria, is associated with an increase in median delta-age. Kruskal-Wallis test was used to compare complexity groups (*P* < 0.001).

The mean AI-ECG estimated age of the cohort was 52.3 ± 16.6 years, with a mean delta-age of 13.2 (±14.6) years indicating a higher AI-ECG estimated than the chronologic age in most patients. The scatter plot of AI-ECG estimated age vs chronologic age in the entire cohort and stratified by CHD complexity is presented in Figure 1. The frequency distribution of delta-age in the entire cohort is presented in Supplemental Figure 1. The mean delta-age increased with higher CHD complexity (Figure 2; severe CHD, delta-age of 17.1 ± 18.2 years; moderate CHD, 12.7 ± 13.6 years; simple ACHD 7.7 ± 11.5 years). The multivariable logistic regression analysis identified several factors independently associated with delta-age >1 SD from mean including male sex, increasing CHD complexity, increasing New York Heart Association class, atrial arrhythmia, smoking, chronic kidney disease, cirrhosis, anemia, and polycythemia (Table 2). The pseudo R^2^ for the model was 0.10.

**Table 2:** Multivariable Logistic Regression Model of Clinical Variables Associated With Delta-Age Greater Than 1 Standard Deviation From Mean Delta-Age

	OR (95% CI)	*P* Value
Male	0.76 (0.66-0.85)	<0.001
CHD complexity		
Simple CHD	Reference	
Moderate CHD	1.95 (1.53-2.51)	<0.001
Severe CHD	2.61 (1.97-3.46)	<0.001
NYHA functional class		
I	Reference	
II	1.44 (1.24-1.67)	<0.001
III	1.90 (1.59-2.28)	<0.001
IV	1.83 (1.22-2.75)	0.004
Atrial arrhythmia	1.74 (1.52-2.00)	<0.001
Sustained ventricular tachycardia	1.02 (0.69-1.48)	0.94
Hypertension	0.92 (0.79-1.06)	0.23
Type 2 diabetes mellitus	0.81 (0.64-1.03)	0.08
Smoking	1.17 (1.00-1.37)	0.04
Coronary artery disease	0.94 (0.72-1.23)	0.68
Chronic kidney disease	1.63 (1.21-2.19)	0.001
Cirrhosis	1.60 (1.01-2.57)	0.05
Stroke	1.13 (0.88-1.45)	0.35
Anemia	1.62 (1.42-1.85)	<0.001
Polycythemia	0.62 (0.51-0.77)	<0.001

Pseudo R^2^ value for the model was 0.1.

CHD = congenital heart disease; CI = confidence interval.

**Figure 3:**
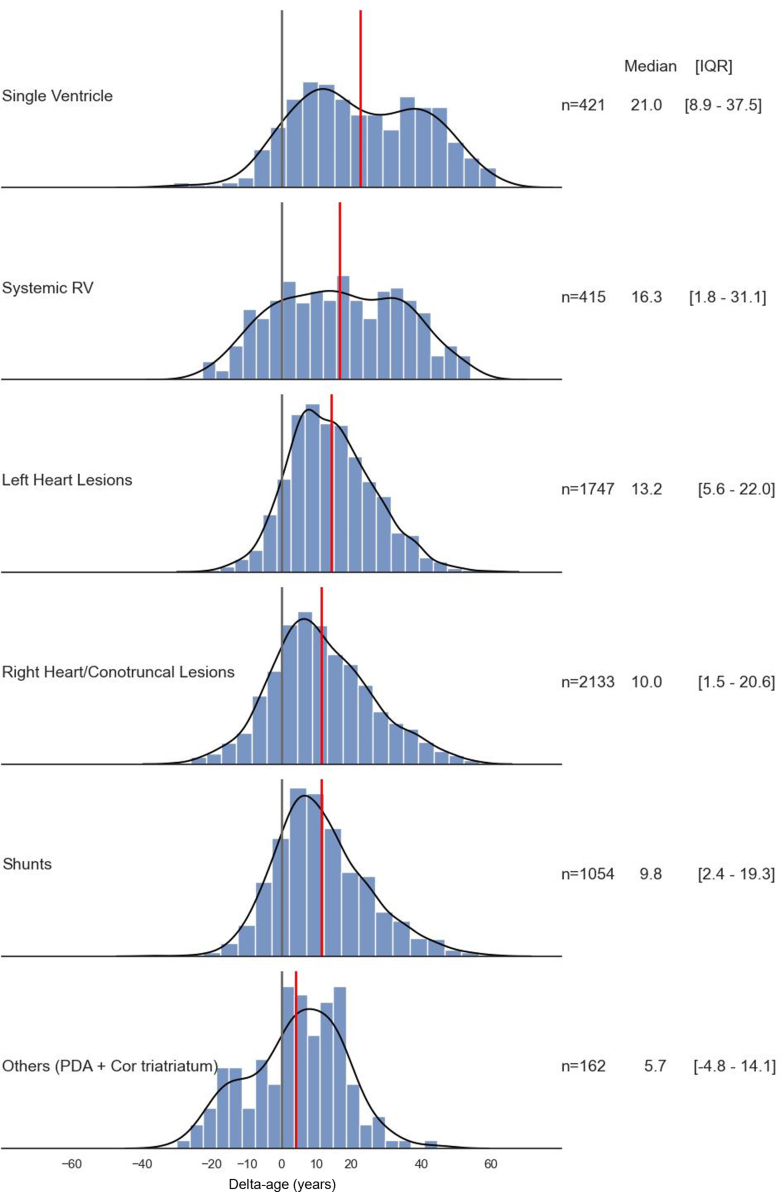
Delta-age Distribution by Congenital Heart Disease Lesion Distribution of delta-age within specific congenital heart disease lesion types. The median value for delta-age is represented by the red line. The gray line represents delta-age of zero, which would be the expected median delta-age for a group of adults in the general population. Left heart lesions included obstructive left heart lesions—coarctation of aorta, mitral stenosis, valvular, supravalvular and subvalvular aortic stenosis. Right heart/conotruncal lesions included Tetralogy of Fallot, pulmonary stenosis, pulmonary atresia, double-outlet right ventricle, truncus arteriosus, Ebstein anomaly, and transposition of great arteries with arterial switch or Rastelli repair. Shunt lesions included isolated ostium secundum or ostium primum atrial septal defects, and sinus venosus defects, complete AV canal defect, partial or total anomalous pulmonary venous return, and isolated ventricular septal defect. Other lesions included isolated patent ductus arteriosus and cor triatriatum. AV = atrioventricular; PDA = patent ductus arteriosus; RV = right ventricle.

The delta-age was estimated in common CHD lesions and presented in Figure 3. Patients with single ventricle had the highest median delta-age of 21.0 (IQR: 8.9-37.5) years. This was followed by patients with systemic right ventricle (median delta-age of 16.3 years, IQR: 1.8-31.1 years) and left heart anomalies (median delta-age 13.2 years, IQR 5.6-22.0 years). Patients with right heart/conotruncal lesions and isolated intracardiac shunts had a similar median delta-age of 10.0 years (IQR: 1.5-20.6) and 9.8 years (IQR: 2.4-19.3), respectively. Higher CHD complexity was again associated with a higher delta-age.

### Delta-age as a predictor of mortality

**Figure 4:**
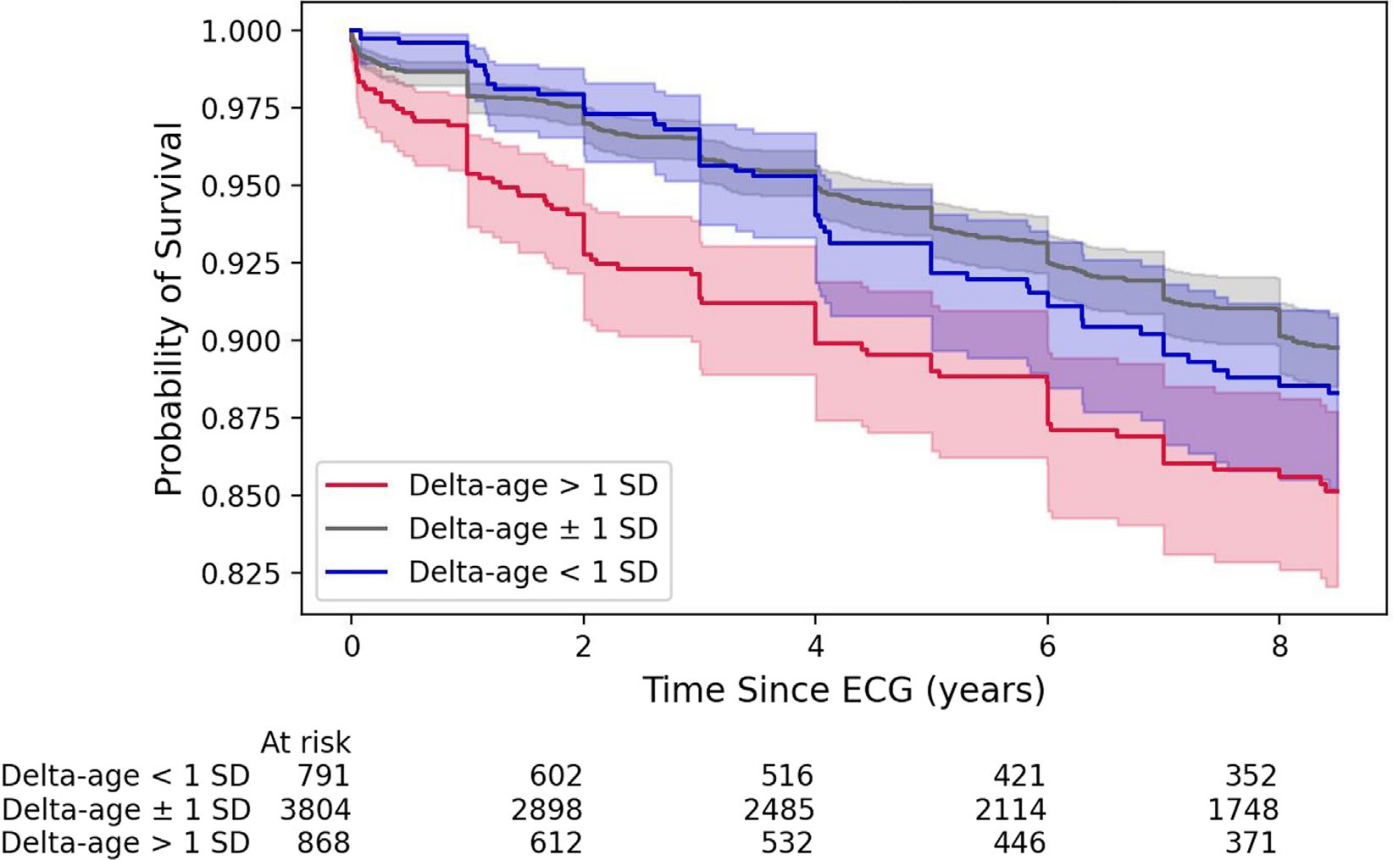
Kaplan-Meier Survival Curve Stratified by Deviation of Delta-Age From the Mean All-cause mortality was significantly different between patients with AI-ECG age greater than or less than one standard deviation from the mean (*P* < 0.005 for log-rank test). Pairwise comparisons: 1) delta-age <1 SD vs within 1 SD of mean was not statistically significant; 2) delta-age <1 SD vs delta-age >1 SD, *P* < 0.005; 3) delta-age >1 SD vs within 1 SD of mean, *P* < 0.005.

**Figure 5:**
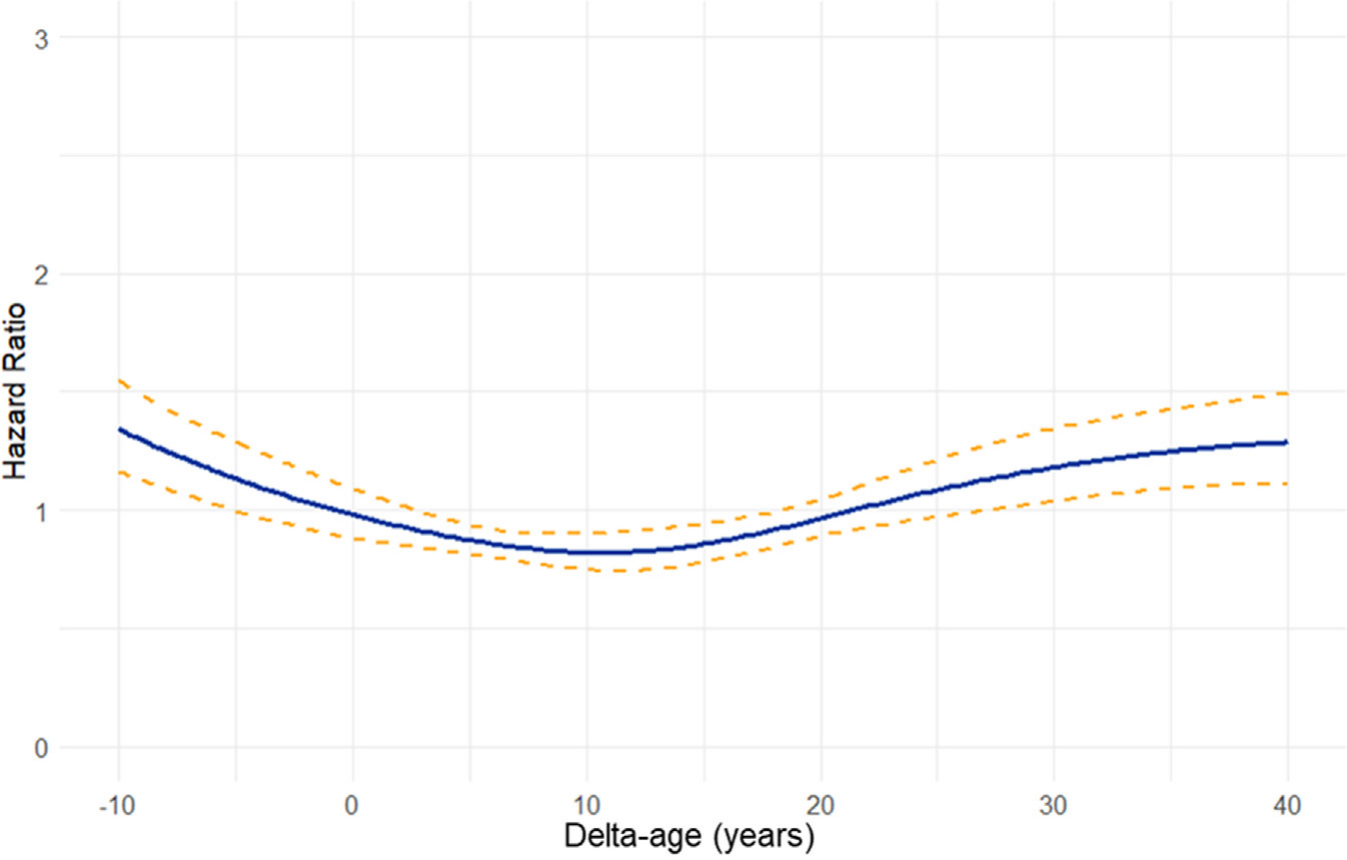
Spline Regression Model of Relationship Between Mortality and Delta-Age Among adults with congenital heart disease, spline regression modeling shows an increased risk of mortality with increasing delta-age.

During a median follow-up of 6.4 years (25th-75th per centile: 1.58-13.7 years), 839 (14.5%) patients died. Mortality rate was 1.78 per 100 person-years of follow-up. Kaplan-Meier analysis of time to death was analyzed in individuals with a delta-age within 1 SD from the mean delta-age and greater than or lesser than 1 SD from mean (Figure 4). Individuals with a delta-age exceeding 1 SD above the mean were at a higher risk of death than those within 1 SD (*P* < 0.005) and those with delta-age <1 SD from mean (*P* < 0.005). The relationship between delta-age and all-cause mortality was evaluated using spline regression models. The Cox proportional hazards model with natural splines revealed a nonlinear association between delta-age and mortality risk. As depicted in Figure 5, both large negative and large positive delta-age were associated with increased mortality risk.

Multivariable analysis of predictors of mortality during follow-up is presented in Table 3. After adjusting for chronologic age, CHD complexity, and other clinically relevant variables, the delta-age was associated with a statistically significant increase in mortality risk (HR: 1.06 [95% CI: 1.03-1.09] per 5-year increment in delta-age, *P* < 0.005). Adjusting for the same variables, 5-year increment in delta-age was also associated with increased risk of 3- and 5-year mortality with HRs of 1.22 (95% CI: 1.15-1.30) and 1.17 (95% CI: 1.11-1.22), respectively. When considered as a categorical variable, delta-age >1 SD from the mean (ie, delta-age >27.8 years) had an increased risk of all-cause mortality compared to the rest of the cohort (HR: 1.67 [95% CI: 1.37-2.05], *P* < 0.005) after adjusting for chronological age, CHD complexity, and other clinically relevant variables (Supplemental Table 1).

**Table 3:** Multivariable Cox Proportional Hazards Model of Predictors of Mortality in Adults With Congenital Heart Disease

	Univariable Model		Multivariable Model	
	HR (95% CI)	*P* Value	HR (95% CI)	*P* Value
Chronological age, per 5-y increase	1.21 (1.18-1.24)	<0.005	1.24 (1.20-1.28)	<0.005
Male	1.09 (0.94-1.27)	0.24	1.10 (0.95-1.27)	0.21
History of atrial arrhythmia	2.22 (1.91-2.59)	<0.005	1.32 (1.14-1.53)	<0.005
History of sustained VT	1.88 (1.31-2.72)	<0.005	1.11 (0.78-1.57)	0.56
CHD complexity				
Mild (reference group)	-		-	
Moderate	1.12 (0.82-1.54)	0.48	1.26 (0.92-1.72)	0.14
Severe	2.28 (1.64-3.17)	<0.005	3.48 (2.49-4.87)	<0.005
NYHA functional class				
I (Reference group)	-		-	
II	1.39 (1.14-1.68)	<0.005	1.32 (1.11-1.58)	<0.005
III	2.12 (1.73-2.59)	<0.005	1.60 (1.31-1.95)	<0.005
IV	3.78 (2.67-5.35)	<0.005	2.86 (2.03-4.02)	<0.005
Hypertension	1.40 (1.20-1.64)	<0.005	0.97 (0.82-1.14)	0.68
Type 2 diabetes mellitus	1.96 (1.58-2.42)	<0.005	1.22 (0.98-1.52)	0.07
History of smoking	1.34 (1.12-1.61)	<0.005	1.13 (0.97-1.37)	0.10
Coronary artery disease	2.05 (1.62-2.58)	<0.005	1.08 (0.86-1.36)	0.50
Chronic kidney disease	4.08 (3.26-5.11)	<0.005	2.13 (1.69-2.68)	<0.005
Cirrhosis	4.81 (3.29-7.04)	<0.005	2.99 (2.03-4.40)	<0.005
Stroke	1.90 (1.47-2.46)	<0.005	1.39 (1.09-1.77)	0.01
Anemia	1.80 (1.54-2.11)	<0.005	1.59 (1.36-1.85)	<0.005
Polycythemia	1.56 (1.26-1.94)	<0.005	1.39 (1.11-1.73)	<0.005
Delta-age (per 5-y increase)	1.00 (0.98-1.03)	0.54	1.06 (1.03-1.09)	<0.005

## Discussion

Applying a previously validated model of AI-ECG-based age estimation, we present evidence for accelerated biologic aging in ACHD, with the difference between AI-ECG estimated age and chronologic age—“delta-age”—increasing with CHD complexity. Reflecting the presence of both cardiac and extracardiac disease states, every 5-year increase in delta-age was associated with a 6% increase in all-cause mortality. Thus, delta-age may serve as a novel biomarker that can be used in clinical practice as a dynamic risk-assessment tool that identifies ACHD at risk of adverse outcomes (Central Illustration).

**Central Illustration:**
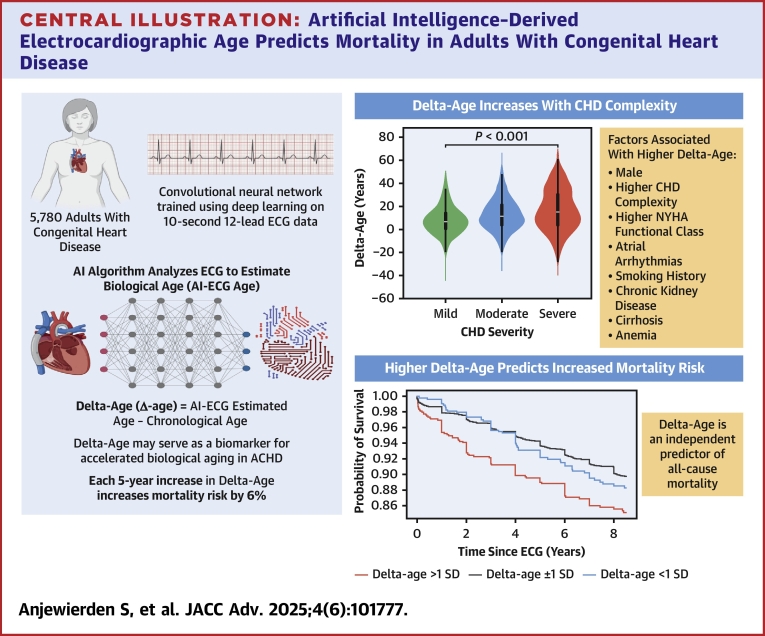
Artificial Intelligence–Derived Electrocardiographic Age Predicts Mortality in Adults With Congenital Heart Disease Delta-age (defined as the difference between AI-ECG-estimated age and chronological age) was evaluated in adults with congenital heart disease. Increasing congenital heart disease complexity was associated with higher delta-age. Among adults with congenital heart disease, higher delta-age was independently associated with an increased mortality risk.

Even as medical advances have improved survival in children born with CHD, ACHD accumulate a range of cardiovascular and extracardiac comorbidities across the life span. ACHD report higher incidence and earlier onset of heart failure, arrhythmias, myocardial infarction, stroke, diabetes, and metabolic syndrome than age-matched individuals without CHD.^2^^,^^11^, ^12^, ^13^, ^14^ Mortality risk in ACHD is 3 times greater than that in age-matched adults without CHD, including a 7 to 10 times greater risk in those with moderate to severe CHD complexity.^2^ Hence, there is a need for well-validated and widely available markers of biological resilience and age in ACHD.

Biological age has been proposed as a measure of an individual's physiologic state and is influenced by genomic, epigenetic, cellular, and environmental factors, in addition to disease states and treatments.^6^ Several methods have been described in the general population to measure biological age including DNA methylation levels, telomere length, and omics-based biomarker panels including proteomics and metabolomics.^15^, ^16^, ^17^, ^18^ However, many of these biomarkers are not routinely obtained in clinical practice, and there is limited experience with these techniques in ACHD. Hence, there is a need for well-validated and widely available markers of biological age in ACHD.

We have previously reported a convoluted neural network to predict age from ECG in adults in the general population with a mean absolute error of 6.9 ± 5.6 years and good correlation with chronological age (r = 0.84).^4^ High delta-age was associated with greater all-cause and cardiovascular mortality in the general population.^5^ Furthermore, patients with a delta-age >7 years had more cardiovascular comorbidities such as atrial fibrillation, coronary artery disease, and hypertension.^4^^,^^19^ In addition to these cardiac disease phenotypes, delta-age was also associated with cardiac specific gene loci including Titin, SCN5A, and Lamin A.^20^^,^^21^ Thus, AI-ECG estimated age correlates with multidimensional markers of cardiovascular health, providing biologic plausibility to its ability to predict cardiovascular outcomes.

In this investigation, we demonstrate that the AI-ECG algorithm applied to the ACHD cohort consistently estimated the age to be significantly higher than the chronological age, with a larger delta observed in those with higher CHD complexity. The 12-lead ECG is influenced by both the underlying CHD and its sequelae including ventricular hypertrophy and dysfunction, atrial enlargement, conduction abnormalities, and arrhythmias, which accumulate over the life span. In addition, we have previously reported that extra-cardiac conditions such as liver cirrhosis can also be accurately detected by CNN applied to ECG.^22^ Hence, the AI interpretation of ECG may reflect multisystem interactions between the heart and other organs, which, in turn, influence biologic age and outcomes.

We propose that AI-ECG-estimated age may serve as a novel marker of biological age that identifies individuals at higher risk of adverse outcomes. Children and adults born with a single ventricle circulation have been shown to have accelerated epigenetic aging that mirrors the premature aging phenotype recognized clinically as early-onset neurocognitive impairment, sarcopenia, exercise intolerance, renal dysfunction, and liver fibrosis.^23^ While ACHD are prone to premature aging, it is evident that biologic aging is not linear. Further studies are required to assess the value of AI-ECG-estimated age as a marker of subclinical changes. In contrast to DNA studies that are inaccessible and unaffordable to many, ECG is a low cost, rapid, and simple test that can be used to follow ACHD patients longitudinally to identify those at increased risk of complications. The finding that patients with single ventricle had the highest delta-age of 21.0 (IQR: 8.9-37.5) years suggests this patient group may be an important focus of future studies examining the value of AI-ECG for guiding early interventions directed not only to reduce morbidity and mortality in ACHD but also to slow biologic aging.

### Study Limitations

This investigation has several limitations. The cohort consisted of predominantly adults with moderate and severe CHD complexity, reflective of a tertiary referral practice. Although simple CHD is underrepresented in this cohort, we believe that the cohort represents a challenging population where biological age assessment is most likely to be beneficial. We adjusted for several known variables that could affect mortality in ACHD. We acknowledge that there may be other confounders that affect this outcome that may not have been accounted for. Given the black-box nature of the CNN algorithm, the specific ECG features that lead to a particular output remain unknown. While “explainability” is desirable for CNN models in the future, the significant clinical associations demonstrated here should be taken into consideration when evaluating the utility of these models. Future studies examining edge cases would provide additional insights into the model's performance and potential limitations. Finally, we propose that the AI-ECG-estimated age may be a marker of biological ageing, but whether this represents ageing independent of other cardiovascular factors is unknown. More investigation is required to establish whether AI-ECG-estimated age correlates with other well-established markers of aging such as DNA methylation clocks and telomere length.

In conclusion, AI-ECG-estimated delta-age is a potential biomarker for accelerated biological aging, increasing with CHD complexity. The difference between AI-ECG and chronological age is an independent predictor of all-cause mortality in ACHD. Incorporating AI-ECG-estimated age into clinical practice holds potential for improving risk stratification and understanding the factors influencing biologic aging in ACHD.

Perspectives **COMPETENCY IN PATIENT CARE:** Care of ACHD includes clinical evaluation and regular cardiac testing. An important aspect of their care includes management of heart failure and minimizing risk of future complications. Utilizing AI-estimated age and delta-age may serve as an easily accessible biomarker to identify early signs of worsening heart disease which may enable providers to escalate care, make medication changes, or perform additional testing needed to improve clinical outcomes.

**TRANSLATIONAL OUTLOOK:** Prospective studies and multicenter validation are needed to further characterize the association between delta-age and clinical outcomes in ACHD.

## Funding support and author disclosures

Dr Anjewierden has received support from the 10.13039/100000002NIH StARR Resident Investigator Award for this project (NIH 5R38HL150086-02). The NIH had no role in the design and conduct of the study. Dr Attia has ownership interest in Xai.health and is an advisor for Anumana.ai and AliveCor. Dr Lopez-Jimenez is an advisor for Anumana; receives royalties/patent beneficiary from Anumana; is a consultant for Kento; is an advisor for Novo Nordisk and Wiseacre. Dr Friedman is associated with Anumana, Eko Health, and AliveCor. Dr Madhavan has received research funding (as a PI or named investigator) from 10.13039/100008497Boston Scientific and is a researcher for Biotronik Inc. All other authors have reported that they have no relationships relevant to the contents of this paper to disclose.
