# Supplementary material for: Artificial Intelligence–Derived Electrocardiographic Age Predicts Mortality in Adults With Congenital Heart Disease
Source: JACC Adv. 2025 May 14;4(6):101777. doi: 10.1016/j.jacadv.2025.101777 (PMC12144430; doi:10.1016/j.jacadv.2025.101777)
Supplement: Supplementary data [file mmc1.pdf]

Supplementary material

Supplementary Figure. Distribution of the delta-age among all adults with congenital heart disease

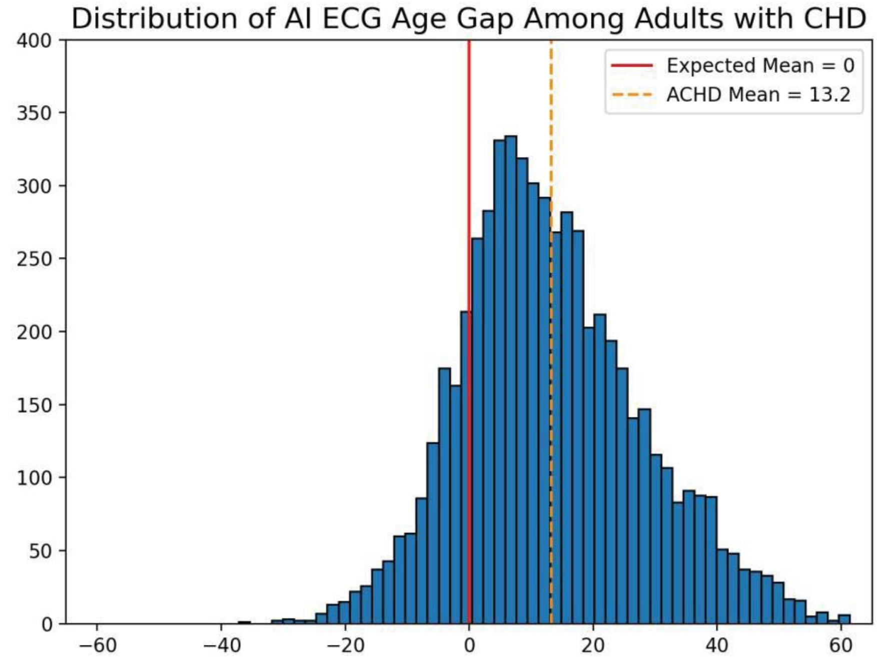

Supplementary Table 1. Multivariable Cox proportional hazards model of predictors of mortality in adults with congenital heart disease evaluating delta-age by standard deviations from the mean delta-age.

	Univariable Model		Multivariable Model	
	HR (95% CI)	p value	HR (95% CI)	p value
Chronological age, per 5-year increase	1.21 (1.18 – 1.24)	< 0.005	1.24 (1.20 – 1.28)	< 0.005
Male sex	1.09 (0.94 – 1.27)	0.24	1.09 (0.95 – 1.26)	0.23
History of atrial arrhythmia	2.22 (1.91 – 2.59)	<0.005	1.32 (1.14 – 1.53)	<0.005
History of sustained VT	1.88 (1.31 –2.72)	<0.005	1.14 (0.81 – 1.62)	0.45
CHD complexity				
Mild (Reference group)	-		-	
Moderate	1.12 (0.82 – 1.54)	0.48	1.28 (0.94 – 1.75)	0.12
Severe	2.28 (1.64 – 3.17)	<0.005	3.49 (2.49 – 4.89)	<0.005
NYHA Class				
I (Reference group)	-		-	
II	1.39 (1.14 – 1.68)	<0.005	1.35 (1.13 – 1.62)	<0.005
III	2.12 (1.73 – 2.59)	<0.005	1.68 (1.38 – 2.04)	<0.005
IV	3.78 (2.67 – 5.35)	<0.005	2.86 (2.03 – 4.03)	<0.005
Hypertension	1.40 (1.20 – 1.64)	<0.005	0.98 (0.83 – 1.15)	0.80
Type 2 diabetes mellitus	1.96 (1.58 – 2.42)	<0.005	1.20 (0.97 – 1.49)	0.10
History of smoking	1.34 (1.12 –1.61)	<0.005	1.17 (0.98 – 1.39)	0.08
Coronary artery disease	2.05 (1.62 – 2.58)	<0.005	1.09 (0.87 – 1.37)	0.46
Chronic kidney disease	4.08 (3.26 – 5.11)	<0.005	2.15 (1.71 – 2.71)	<0.005
Cirrhosis	4.81 (3.29 – 7.04)	<0.005	3.13 (2.13 – 4.61)	<0.005
History of stroke	1.90 (1.47 – 2.46)	<0.005	1.36 (1.07 – 1.73)	0.01
Anemia	1.80 (1.54 – 2.11)	<0.005	1.61 (1.38 – 1.87)	<0.005
Polycythemia	1.56 (1.26 – 1.94)	<0.005	1.34 (1.08 – 1.68)	0.01
Delta-age				
>1 SD above mean	1.45 (1.19 – 1.76)	<0.005	1.67 (1.37 – 2.05)	<0.005
Within 1 SD (Reference group)	-		-	
> 1 SD below mean	1.49 (1.22 – 1.82)	<0.005	1.05 (0.87 – 1.26)	0.65

STROBE Statement—checklist of items that should be included in reports of observational studies

	Item No.	Recommendation	Page No.	Relevant text from manuscript
**Title and abstract**	1	(*a*) Indicate the study's design with a commonly used term in the title or the abstract	2	…12-lead ECGs in a cohort of adults (age >18 years)…
		(*b*) Provide in the abstract an informative and balanced summary of what was done and what was found	2-3	See abstract results
**Introduction**				
Background/rationale	2	Explain the scientific background and rationale for the investigation being reported	4	
Objectives	3	State specific objectives, including any prespecified hypotheses	5	We hypothesized that AI enhanced interpretation of ECG in ACHD will yield an estimation of the biological age that correlates with comorbidities and mortality
**Methods**				
Study design	4	Present key elements of study design early in the paper	4-5	
Setting	5	Describe the setting, locations, and relevant dates, including periods of recruitment, exposure, follow-up, and data collection	4-6	
Participants	6	(*a*) *Cohort study*—Give the eligibility criteria, and the sources and methods of selection of participants. Describe methods of follow-up<br>*Case-control study*—Give the eligibility criteria, and the sources and methods of case ascertainment and control selection. Give the rationale for the choice of cases and controls<br>*Cross-sectional study*—Give the eligibility criteria, and the sources and methods of selection of participants	4-5	This retrospective cohort study included adult patients (age >18 years) evaluated at the Mayo Clinic for Adult Congenital Heart Disease Clinic between the years of 1992 to 2023.

		(*b*) *Cohort study*—For matched studies, give matching criteria and number of exposed and unexposed<br><br>*Case-control study*—For matched studies, give matching criteria and the number of controls per case	N/A
Variables	7	Clearly define all outcomes, exposures, predictors, potential confounders, and effect modifiers. Give diagnostic criteria, if applicable	5-6
Data sources/ measurement	8*	For each variable of interest, give sources of data and details of methods of assessment (measurement). Describe comparability of assessment methods if there is more than one group	*5-6*
Bias	9	Describe any efforts to address potential sources of bias	6
Study size	10	Explain how the study size was arrived at	4-5

Continued on next page

Quantitative variables	11	Explain how quantitative variables were handled in the analyses. If applicable, describe which groupings were chosen and why	6	Delta-age was evaluated both as a continuous variable and as a categorical variable. For the latter, delta-age was categorized into three groups after computing the mean and SD of the delta-age for the entire cohort. The groups included those with delta-age <1 SD from mean, within ±1 SD, and >1 SD from the mean.
Statistical methods	12	(*a*) Describe all statistical methods, including those used to control for confounding	6	
		(*b*) Describe any methods used to examine subgroups and interactions	6-7	
		(*c*) Explain how missing data were addressed	5	Those that had missing data (either clinical information or ECG data) were excluded
		(*d*) *Cohort study*—If applicable, explain how loss to follow-up was addressed<br>*Case-control study*—If applicable, explain how matching of cases and controls was addressed<br>*Cross-sectional study*—If applicable, describe analytical methods taking account of sampling strategy	n/a	
		(*e*) Describe any sensitivity analyses	n/a	
**Results**				
Participants	13*	(a) Report numbers of individuals at each stage of study—eg numbers potentially eligible, examined for eligibility, confirmed eligible, included in the study, completing follow-up, and analysed	7	
		(b) Give reasons for non-participation at each stage	7	
		(c) Consider use of a flow diagram	n/a	
Descriptive data	14*	(a) Give characteristics of study participants (eg demographic, clinical, social) and information on exposures and potential confounders	7 (and table 1)	

		(b) Indicate number of participants with missing data for each variable of interest	n/a	
		(c) *Cohort study*—Summarise follow-up time (eg, average and total amount)	8	During a median follow-up of 6.4 years (25th – 75th centile 1.58 – 13.7 years), 839 (14.5%) patients died
Outcome data	15*	*Cohort study*—Report numbers of outcome events or summary measures over time	8	839 (14.5%) patients died. Mortality rate was 1.78 per 100 person-years of follow-up
		Case-control study—Report numbers in each exposure category, or summary measures of exposure		
		Cross-sectional study—Report numbers of outcome events or summary measures		
Main results	16	(*a*) Give unadjusted estimates and, if applicable, confounder-adjusted estimates and their precision (eg, 95% confidence interval). Make clear which confounders were adjusted for and why they were included	8-9	After adjusting for chronologic age, CHD complexity, and other clinically relevant variables, the delta-age was associated with a statistically significant increase in mortality risk (HR 1.06 (1.03 – 1.09) per 5-year increment in delta-age, p<0.005).
		(*b*) Report category boundaries when continuous variables were categorized	9	When considered as a categorical variable, delta-age >1SD from the mean (i.e. delta-age > 27.8 years) had an increased risk of all-cause mortality compared to the rest of the cohort (HR 1.67 [1.37 – 2.05], p <0.005) after adjusting for chronological age, CHD complexity and other clinically relevant variables
		(*c*) If relevant, consider translating estimates of relative risk into absolute risk for a meaningful time period		

Continued on next page

Other analyses	17	Report other analyses done—eg analyses of subgroups and interactions, and sensitivity analyses	7-9
**Discussion**			
Key results	18	Summarise key results with reference to study objectives	9
Limitations	19	Discuss limitations of the study, taking into account sources of potential bias or imprecision. Discuss both direction and magnitude of any potential bias	11-12
Interpretation	20	Give a cautious overall interpretation of results considering objectives, limitations, multiplicity of analyses, results from similar studies, and other relevant evidence	9-11
Generalisability	21	Discuss the generalisability (external validity) of the study results	10-11
**Other information**			
Funding	22	Give the source of funding and the role of the funders for the present study and, if applicable, for the original study on which the present article is based	12

*Give information separately for cases and controls in case-control studies and, if applicable, for exposed and unexposed groups in cohort and cross-sectional studies.

Note: An Explanation and Elaboration article discusses each checklist item and gives methodological background and published examples of transparent reporting. The STROBE checklist is best used in conjunction with this article (freely available on the Web sites of PLoS Medicine at http://www.plosmedicine.org/, Annals of Internal Medicine at http://www.annals.org/, and Epidemiology at http://www.epidem.com/). Information on the STROBE Initiative is available at www.strobe-statement.org.

STROBE Statement—checklist of items that should be included in reports of observational studies

	Item No.	Recommendation	Page No.	Relevant text from manuscript
**Title and abstract**	1	(*a*) Indicate the study's design with a commonly used term in the title or the abstract	2	…12-lead ECGs in a cohort of adults (age >18 years)…
		(*b*) Provide in the abstract an informative and balanced summary of what was done and what was found	2-3	See abstract results
**Introduction**				
Background/rationale	2	Explain the scientific background and rationale for the investigation being reported	4	
Objectives	3	State specific objectives, including any prespecified hypotheses	5	We hypothesized that AI enhanced interpretation of ECG in ACHD will yield an estimation of the biological age that correlates with comorbidities and mortality
**Methods**				
Study design	4	Present key elements of study design early in the paper	4-5	
Setting	5	Describe the setting, locations, and relevant dates, including periods of recruitment, exposure, follow-up, and data collection	4-6	
Participants	6	(*a*) *Cohort study*—Give the eligibility criteria, and the sources and methods of selection of participants. Describe methods of follow-up<br>*Case-control study*—Give the eligibility criteria, and the sources and methods of case ascertainment and control selection. Give the rationale for the choice of cases and controls<br>*Cross-sectional study*—Give the eligibility criteria, and the sources and methods of selection of participants	4-5	This retrospective cohort study included adult patients (age >18 years) evaluated at the Mayo Clinic for Adult Congenital Heart Disease Clinic between the years of 1992 to 2023.
		(*b*) *Cohort study*—For matched studies, give matching criteria and number of exposed and unexposed<br>*Case-control study*—For matched studies, give matching criteria and the number of controls per case	N/A	

Variables	7	Clearly define all outcomes, exposures, predictors, potential confounders, and effect modifiers. Give diagnostic criteria, if applicable	5-6
Data sources/ measurement	8*	For each variable of interest, give sources of data and details of methods of assessment (measurement). Describe comparability of assessment methods if there is more than one group	*5-6*
Bias	9	Describe any efforts to address potential sources of bias	6
Study size	10	Explain how the study size was arrived at	4-5

Continued on next page

Quantitative variables	11	Explain how quantitative variables were handled in the analyses. If applicable, describe which groupings were chosen and why	6	Delta-age was evaluated both as a continuous variable and as a categorical variable. For the latter, delta-age was categorized into three groups after computing the mean and SD of the delta-age for the entire cohort. The groups included those with delta-age <1 SD from mean, within ±1 SD, and >1 SD from the mean.
Statistical methods	12	(*a*) Describe all statistical methods, including those used to control for confounding	6	
		(*b*) Describe any methods used to examine subgroups and interactions	6-7	
		(*c*) Explain how missing data were addressed	5	Those that had missing data (either clinical information or ECG data) were excluded
		(*d*) *Cohort study*—If applicable, explain how loss to follow-up was addressed<br>*Case-control study*—If applicable, explain how matching of cases and controls was addressed<br>*Cross-sectional study*—If applicable, describe analytical methods taking account of sampling strategy	n/a	
		(*e*) Describe any sensitivity analyses	n/a	
**Results**				
Participants	13*	(a) Report numbers of individuals at each stage of study—eg numbers potentially eligible, examined for eligibility, confirmed eligible, included in the study, completing follow-up, and analysed	7	
		(b) Give reasons for non-participation at each stage	7	
		(c) Consider use of a flow diagram	n/a	
Descriptive data	14*	(a) Give characteristics of study participants (eg demographic, clinical, social) and information on exposures and potential confounders	7 (and table 1)	
		(b) Indicate number of participants with missing data for each variable of interest	n/a	
		(c) *Cohort study*—Summarise follow-up time (eg, average and total amount)	8	During a median follow-up of 6.4 years (25th – 75th centile 1.58 –

				13.7 years), 839 (14.5%) patients died
Outcome data	15*	*Cohort study*—Report numbers of outcome events or summary measures over time	8	839 (14.5%) patients died. Mortality rate was 1.78 per 100 person-years of follow-up
		Case-control study—Report numbers in each exposure category, or summary measures of exposure		
		Cross-sectional study—Report numbers of outcome events or summary measures		
Main results	16	(*a*) Give unadjusted estimates and, if applicable, confounder-adjusted estimates and their precision (eg, 95% confidence interval). Make clear which confounders were adjusted for and why they were included	8-9	After adjusting for chronologic age, CHD complexity, and other clinically relevant variables, the delta-age was associated with a statistically significant increase in mortality risk (HR 1.06 (1.03 – 1.09) per 5-year increment in delta-age, p<0.005).
		(*b*) Report category boundaries when continuous variables were categorized	9	When considered as a categorical variable, delta-age >1SD from the mean (i.e. delta-age > 27.8 years) had an increased risk of all-cause mortality compared to the rest of the cohort (HR 1.67 [1.37 – 2.05], p <0.005) after adjusting for chronological age, CHD complexity and other clinically relevant variables
		(*c*) If relevant, consider translating estimates of relative risk into absolute risk for a meaningful time period		

Continued on next page

Other analyses	17	Report other analyses done—eg analyses of subgroups and interactions, and sensitivity analyses	7-9
**Discussion**			
Key results	18	Summarise key results with reference to study objectives	9
Limitations	19	Discuss limitations of the study, taking into account sources of potential bias or imprecision. Discuss both direction and magnitude of any potential bias	11-12
Interpretation	20	Give a cautious overall interpretation of results considering objectives, limitations, multiplicity of analyses, results from similar studies, and other relevant evidence	9-11
Generalisability	21	Discuss the generalisability (external validity) of the study results	10-11
**Other information**			
Funding	22	Give the source of funding and the role of the funders for the present study and, if applicable, for the original study on which the present article is based	12

*Give information separately for cases and controls in case-control studies and, if applicable, for exposed and unexposed groups in cohort and cross-sectional studies.

Note: An Explanation and Elaboration article discusses each checklist item and gives methodological background and published examples of transparent reporting. The STROBE checklist is best used in conjunction with this article (freely available on the Web sites of PLoS Medicine at http://www.plosmedicine.org/, Annals of Internal Medicine at http://www.annals.org/, and Epidemiology at http://www.epidem.com/). Information on the STROBE Initiative is available at www.strobe-statement.org.
